# Composition and Insecticidal Activity of Essential Oil of *Bacopa caroliniana* and Interactive Effects of Individual Compounds on the Activity

**DOI:** 10.3390/insects11010023

**Published:** 2019-12-27

**Authors:** Tai-Ti Liu, Louis Kuo-Ping Chao, Kai-Siang Hong, Yi-Jhen Huang, Tsung-Shi Yang

**Affiliations:** 1 Department of Food Science, Yuanpei University of Medical Technology, No. 306 Yuanpei Street, Hsinchu 30015, Taiwan; 2 Department of Cosmeceutics, China Medical University, No. 91, Hsueh-Shih Road, Taichung 40402, Taiwan

**Keywords:** *Bacopa caroliniana*, essential oil, insecticidal activity, rice weevil, interactive effect

## Abstract

*Bacopa caroliniana* (BC) is a perennial creeping herb and popular aquarium plant. This plant is easily cultivated; consequently, it has the potential to be a raw material which is readily available for mass production if it contains useful bioactive substances. The information about the functionality of this plant has been very limited. Therefore, the aims of this research were to analyze the composition of the essential oil (EO) of BC and to study its insecticidal effect on rice weevils. Moreover, the interactive effects of active compounds of the EO on this activity were also investigated. A total of 18 volatile compounds was identified, accounting for ca. 94% of the BC-EO in terms of quantity. Of them, α-terpinolene was the largest compound. The impact of individual volatile compounds on the inhibition of acetylcholine esterase and insecticidal activity were determined. α-Terpinolene exhibited the highest activity on these assays. Both additive and synergistic effects existed in terms of the insecticidal activity. Many compounds found in the BC-EO are widely present in other EOs. Thus, the information obtained from this study is useful for EO-related research, applications in selecting EOs or in seeking the best combination of EOs or individual compounds to achieve efficient insecticidal effects.

## 1. Introduction

Many herbs have been used as food or medicine since ancient times because they contain a wide variety of bioactive phytochemicals, which have been reported to reduce the risk of diseases and promote health [1]. Regarding phytochemicals, essential oils (EOs) are mainly responsible for the aroma of aromatic plants and provide plenty of functionality such as antimicrobial, antioxidant, anti-inflammatory, and pesticide repelling or insecticidal activity.

In regard to insect pests, some are a detrimental threat to stored grains, which may cause the physical, nutritional, and quality deterioration of the grains. Of all the coleopteran pests of stored grains, the most damaging species of storage insects are found in the genus *Sitophilus*. For example, rice weevils (*Sitophilus oryzae* L.) are an internal feeding insect pest that bores into stored grains, causing damage by consuming the grains [2]. The management of agricultural pests has been largely dependent on the use of synthetic chemical pesticides for the field and post-harvest protection of crops. Nevertheless, the indiscriminate use of synthetic pesticides has caused many serious problems such as toxicity to non-target organisms, development of pest resistance and resurgence, and environmental contamination [3]. EOs and their derivatives are considered to be an alternative means of controlling many harmful insects [4]. Many EOs such as *Ruta graveolens* [5], *Cinnamomum tamala* [6], and *Cleistanthus collinus* [7] have been reported to be effective against rice weevils.

*Bacopa caroliniana* (Walt.) B.L. Robins. is a perennial creeping herb. The leaves of this plant are relatively thick and succulent, releasing a smell of lemon if crushed. The flowers are blue, with five petals, and it grows to 50–100 cm. It can be used as an aquarium plant due to its ability to grow in water. In the wild, it grows in bogs or semi-submersed conditions, adapting well if flooded and fully submerged [8]. This plant is easily cultivated because only simple conditions are sufficient for growth. Consequently, it has the potential to be a raw material readily which is available for mass production if it contains useful bioactive substances. The information about the functionality of this plant has been very limited. To the best of our knowledge, only the antimicrobial activity of the plant extract has been reported [9]. The composition of essential oil of BC (BC-EO) has not been investigated. In addition, the insecticidal activity has not been studied either.

Concerning research into the functionality of EOs, most studies have used whole EOs or their major compounds for the investigation [10,11]. Relatively few studies have worked on the interactive effects [12,13]. Thus, the information about the effects of interaction among individual compounds on the functionality of EOs has been limited. Therefore, the aims of this research were to analyze the composition of BC-EO and to study its insecticidal activity on rice weevils. Moreover, the interactive effects of active compounds of the EO on this activity were also investigated.

## 2. Materials and Methods

### 2.1. Materials and Chemicals

The *Bacopa caroliniana* herb was purchased from a local horticulture market (Taichung, Taiwan). The chemicals α-pinene (≥95%), β-pinene (≥96%), *p*-cymene (≥90%), sabinene (≥92%), linalool (≥97%), nerolidol (≥95%), α-terpineol (≥90%), 4-terpineol (≥95%), β-caryophyllene (≥85%), β-caryophyllene oxide (≥95%), 1-octen-3-ol (≥95%), 1,8-cineole (≥90%), α-terpinolene (≥92%), were obtained from K. F. Lings Co. (Taoyuan, Taiwan). Citronellol (≥95%) and α-terpinene (≥90%) was acquired from Alfa Aesar. Myrcene (≥90%) and γ-terpinene (≥97%) were purchased from Acros Organics. Limonene (≥95%), acetylthiocholine iodide, and 5,5-dithio-bis (2-nitrobenzoic acid) were purchased from Sigma-Aldrich Co.

### 2.2. Isolation of Essential Oil

The amount of 200 g of BC herb in a fresh state was cut and placed in a glass flask. Double de-ionized water was added into the flask to make the final volume of 1500 mL. The sample was subjected to hydrodistillation using a Clevenger-type apparatus. The vapor mixture of water and EO produced in the flask passed through a condenser, and then the distillate was collected. The EO in the upper layer of the distillate was taken and dried over anhydrous sodium sulfate.

### 2.3. Analysis of Essential Oil by GC-MS and GC-FID

A volume of 0.5 μL of the BC-EO was injected into a gas chromatograph (GC, model 6890N, Agilent, Palo Alto, CA, USA) equipped with a quadruple mass analyzer (MS, Agilent 5975) or flame ionization detector (FID). Capillary columns (DB-WAX, J &W Scientific, Folsom, CA, USA) were used with helium as a carrier gas at a constant flow of 1 mL/min. The oven temperature was programmed as the following: initial holding at 40 °C for 1 min, then 140 °C (2 min) at 5 °C/min, and 200 °C (30 min) at 3 °C/min. The MS operating parameters were the following: electron impact mode for molecular ionization with a voltage of 70 eV; ion source temperature, 200 °C; total ion scan mode with a scan rate of 4.37 scans/s and a mass scan range of 29–350 *m*/*z.* The amounts of volatiles were quantified by peak areas integrated by a computer system (Chem station^TM^, Agilent Technologies, Palo Alto, CA, USA). The volatiles were identified by comparing their mass spectra with those in the library of the MS data system (Wiley 275, G1035A, Agilent Technologies, Palo Alto, CA, USA) and with those of standard compounds. The amounts of individual volatiles of the EO were expressed as percentages of the peak area relative to the total peak area obtained from a flame ionization detector (FID) at 250 °C under the same GC conditions as above.

### 2.4. In Vitro AChE Inhibition Assay

Untreated whole rice weevil adults (0.1 g) were homogenized with 1 mL phosphate buffer (pH 7.4). The homogenate was centrifuged at 8775 g for 20 min at 0 °C, and the supernatant was used as crude acetylcholine esterase (AChE). The inhibition of AChE was determined by the colorimetric method of Ellman et al. [14] using acetylthiocholine iodide (ATChI) at 2.5 mM as the substrate. Enzyme aliquots (20 μL) and 5,5-dithio-bis (2-nitrobenzoic acid) (20 μL, 4 mM) were added to 0.1M phosphate buffer (pH 7.4, 120 μL), and the EO active compounds (20 μL) prepared in absolute ethanol were added to this mixture. The addition of absolute ethanol (20 μL) rather than the active compound was used in the control treatment. All mixtures were incubated at 35 °C for 15 min, and the reactions were started by adding ATChI (20 μL), with absorbance measured at 412 nm using a microplate reader (EL800, Bio Tek).

### 2.5. Toxicity Bioassay of Rice Weevils

The rice weevils from our culture source without exposure to any known insecticide were reared on polished rice in a plastic container (27 cm × 19 cm × 11 cm) at 30 ± 2 °C, and 75 ± 3% humidity for 20 days. The adult weevils of a similar size were selected for later experiments. A filter paper toxicity bioassay was applied to evaluate the insecticidal activity on the insects. First, the samples were dissolved in acetone with a serious of concentrations. A filter paper (No. 5C, 9 cm, Advantec™) was immersed into the solution for 1 min, and the weight of the sample absorbed in the paper was calculated after the solvent was evaporated for 1 min. The paper was fixed inside the lid of a Petri dish. Twenty insects were placed into the Petri dish before the lid was covered. The Petri dishes were stored in an incubator at 30 ± 2 °C for 24 h, and the survival rate of the insects was calculated compared with the control group. The assay was performed in triplicate.

### 2.6. Interactive Effects of Individual Compounds on Functionality

The compounds with good functionality were selected for investigating the interactive effects. The checkerboard method was employed to quantitatively examine the interactive effect of active compounds via the calculation of the fractional inhibitory concentration (FIC) index: FIC index = FIC_A_ + FIC_B_, where FIC_A_ = the IC_50_ value of compound A in combination/the IC_50_ value of compound A alone, and FIC_B_ = the IC_50_ value of compound B in combination/the IC_50_ value of compound B alone_._ The results were interpreted as synergy (FIC ≤ 0.5), addition (0.5< FIC ≤ 1), indifference (1 < FIC ≤ 4) or antagonism (FIC > 4) [15]. The assays were performed in duplicate and then replicated.

## 3. Results and Discussion

### 3.1. Identification and Composition of Volatile Compounds in BC-EO

Excluding the compounds that were trace in quantity (<0.5 g kg^−1^), 18 compounds accounting for ca. 94% of the total amount of the BC-EO were tentatively identified by GC-MS and then positively identified with the corresponding standard compounds. The composition of the volatile compounds is listed in Table 1. The majority of the compounds belong to terpenes and terpenoids. In terms of the quantity, monoterpene hydrocarbons were the largest group among the identified compounds in the EO. Noticeably, α-terpinolene was the largest compound in the EO.

### 3.2. Impact of Individual Volatile Compounds in BC-EO on Inhibition of Acetylcholine Esterase

Effects of EOs on insects are presumed to interfere with their basic metabolic, biochemical, physiological, and behavioral functions, but little is known about the true mode of action of EOs [3]. The rapid onset of toxic signs suggests a neurotoxic mode of action involving competitive inhibition of acetylcholine esterase (AChE) [17]. Inhibition of AChE causes an accumulation of acetylcholine at the synapses, meaning that the post-synaptic membrane is in a state of permanent stimulation, which results in a general lack of co-ordination in the neuromuscular system and eventual death [18,19]. Therefore, the inhibition of AChE was used to evaluate the effect of BC-EO and its constituents on rice weevils.

From the results in Table 2, α-pinene, limonene, and α-terpinolene showed good AChE inhibitory activity with IC_50_ values < 10 µL mL^−1^, especially α-pinene (1.81 µL mL^−1^) and α-terpinolene (1.1 µL mL^−1^). In contrast to α-pinene, β-pinene did not result in AChE inhibitory activity in our test range. This implies that there seems to be a structural effect between α-pinene and β-pinene on this activity. Similarly, it has been reported that α-pinene is a potent inhibitor of AChE, and β-pinene is inferior to α-pinene [20]. α-Terpinolene has been found to be a good inhibitor of AChE obtained from rice weevils [21]. In our study, α-terpinolene also exhibited the best activity among the test compounds. The IC_50_ values of limonene, 4-terpineol, α-terpineol, and 1-octen-3-ol were in the range of 10-20 µL mL^−1^. Even though these compounds were not as effective as α-pinene or α-terpinolene, they still showed good activity as compared with the other compounds.

In another aspect, 1,8-cineole and linalool exhibited relatively moderate activity, with IC_50_ values in the range of 30–45 µL mL^−1^. In one study, 1,8-cineole, α-pinene, 4-terpineol, and α-terpineol were reported as potent AChE inhibitors, of which 1,8-cineole was strongest. However, linalool is a weak inhibitor; limonene, γ-terpinene, and β-caryophyllene are inactive within the range of test concentrations in the study of Dohi et al. [22] using AChE extracted from electric eels. In another study [23], α-pinene, β-pinene, and limonene show good AChE (rice weevil source) inhibitory activity, of which β-pinene has the most potent activity. Nonetheless, linalool is not effective. Comparatively, in our study, γ-terpinene and β-caryophyllene were not effective, which was similar to the study of Dohi et al. [22] However, 1,8-cineole was moderately active and inferior to limonene. Some variations of the results on AChE inhibition among different studies in literature or between the studies and this research might be ascribed to the differences in enzyme sources, sample preparation, and quantification methods.

The impact of AChE inhibitory activity (EI) was calculated according to the method of Liu and Yang [24]. Briefly, the impact is the ratio of activity of the target compound vs the compound with the least effect and then multiplied by the content (%) of the target compound. When the EI values were compared, some major compounds did not exhibit a marked impact on this activity. For instance, 1,8-cineole, γ-terpinene, and *p*-cymene with the high contents (10–20%) did not show effects proportional to their quantity. In contrast, α-terpinolene, the most abundant compound, had the greatest impact. The EI value of α-terpinolene was considerably high as compared with those of the others because of its highest content and activity. Thus, it can be concluded that α-terpinolene predominantly influences the AChE inhibitory activity of the BC-EO.

### 3.3. Interaction of Individual Volatile Compounds on the Inhibition of Acetylcholine Esterase

Based on the EI values, the compounds (except for linalool) with values >20 were selected for the investigation of interactive effects between the compounds on the AChE inhibition. The results in Table 3 revealed that α-terpinolene had synergistic effects with sabinene, limonene, and α-pinene, but additive effects with 1,8-cineole, 4-terpineol, 1-octen-3-ol, and linalool. These results exhibited a trend that α-terpinolene tended to interact synergistically with the terpenes, but additively with the terpenoids. Sabinene had synergistic effects with limonene and 4-terpineol, but additive effects with 1,8-cineole, α-pinene, 1-octen-3-ol, and linalool. This indicated that sabinene did not show a preference for the terpenes or terpenoids. Regarding limonene, it simply had a synergistic effect with α-pinene but had additive effects with 1,8-cineole, 4-terpineol, 1-octen-3-ol, and limalool. Similarly, except for limonene, α-pinene interacted additively with 1,8-cineole, 4-terpineol, 1-octen-3-ol, and lanalool. Apparently, α-pinene and limonene showed synergistic preference for the terpenes rather than the terpenoids. In another aspect, 1,8-cineole and 1-octen-3-ol had synergistic effects with the terpenoids except for linalool. Apart from sabinene and linalool, 4-terpineol exhibited synergistic effects with all terpenoids. These results suggest that, excluding a few exceptions, the terpenes tend to have synergistic effects with the terpenes, and this trend is also applied to the interaction between terpenoids. When all the EI values of the test compounds are totalized, the value is 8206, which is much smaller than that of the EO (25929). This shows that the great impact of the EO comes from the synergistic effects of some individual compounds as described above.

### 3.4. Insecticidal Impact of Individual Volatile Compounds in BC-EO

The insecticidal effects of the test compounds are listed in Table 2. The compounds with LC_50_ values between 100 and 200 (µg mL^−1^) in ascending order were limonene, linalool, 1-octen-3-ol, and α-terpinolene, of which limonene had the strongest effect. The compounds with IC_50_ values between 200 and 400 (µg mL^−1^) in ascending order were α-terpineol, citronellol, α-pinene, 1,8-cineole, and sabinene. The insecticidal effects of some compounds of many EOs have been reported. For instance, the good insecticidal activity of α-terpinolene, which is the major compound in the EO of *Ocotea longifolia* Kunth., has been found [21]. Linalool showed moderate AChE inhibitory activity but had the second-best effect on the insecticidal activity in this experiment. The reason might be that, except for the AChE inhibitory activity, linalool can also affect the ion transport and release of acetylcholine on the nervous system [25], which may influence the motility of the insects as well. Limonene has been reported to have good fumigant toxicity to rice weevils [4]. The insecticidal activity of 1,8-cineole, p-cymene, α-pinene, and limonene has been compared, and the order was 1,8-cineole > *p*-cymene > α-pinene > limonene [26]. However, some of the results in this study were consistent or inconsistent with the reported data in the literature. For example, limonene had better activity than 1,8-cineole, *p*-cymene, and α-pinene as opposed to the study of Lee et al. [26]. The discrepancy might be caused by the different methods used such as the sample preparation, chamber type, and experimental conditions. For example, the solvent used for dissolving the test compounds was not removed in their study; in contrast, the solvent was removed and the residual contents of the compounds were calculated and then used in this experiment. Therefore, the volatility of compounds and their affinity with the solvent during evaporation may affect the residual contents and subsequent experimental results.

Regarding the insecticidal impact (TI) calculated as EI, α-terpinolene, 1,8-cineole, and γ-terpinene were three most influential compounds on this activity, with TI values of 39.76, 36.2, and 23.79, respectively. These compounds accounted for ca. 62% of the EO; reasonably, they showed proportional influence on the insecticidal activity. The compounds with TI values in the range of 10–20 were sabinene, 4-terpineol, and *p*-cymene in descending order. The result revealed that sabinene and 4-terpineol had greater impact than *p*-cymene, even though their contents were much lower than that of *p*-cymene. The insecticidal activity of limonene, linalool, and 1-octen-3-ol was no less than α-terpinolene, but their impact was less than that of α-terpinolene due to their much lower contents.

When the insecticidal activity and AChE inhibitory activity were compared, generally most compounds showed good correlation between both activities. Namely, the compounds with higher AChE inhibitory activity tended to have higher insecticidal activity. However, some compounds varied. For instance, the AChE inhibitory activity of α-pinene was greater than that of limonene, but an inverse result in the insecticidal activity was observed. Likewise, α-terpinolene had greater AChE inhibitory activity but less insecticidal activity than linalool. It has been reported that some compounds produced strong inhibitory activity towards AChE, but did not show any insecticidal activity [26,27,28]. The reason for this is probably their inability to reach the target site due to a hindrance caused by their topological and physicochemical properties [27].

### 3.5. Interaction of Individual Volatile Compounds on Insecticidal Activity

According to the TI values, the compounds with the values >5 and linalool (its TI value > that of α-terpinolene) were chosen to investigate the interactive effect between the compounds on the insecticidal activity. The results are presented in Table 4. Regarding the terpenes, α-terpinolene had synergistic effects with all test compounds. Sabinene gave synergistic effects with limonene, α-pinene, and 4-terpineol, and linalool, but additive effects with 1,8-cineole and 1-octen-3-ol. Except for 1,8-cineole, limonene showed synergistic effects with sabinene, 4-terpineol, 1-octen-3-ol, and linalool. With respect to the terpenoids, linalool showed synergistic effects with all test compounds probably owing to the dual inhibitory effects described above. 1,8-Cineole exhibited synergistic effects with 4-terpineol, 1-octen-3-ol, and linalool. In addition, 4-terpineol gave a synergistic effect with 1-octen-3-ol. From these results, the activity of the individual compounds and compound types seems to have some influences. If the activity of individual compounds is strong enough, the interaction tends to be synergistic, such as α-terpinolene or linalool with all test compounds. In another case, the activity of sabinene was moderate but showed a synergistic effect with limonene, which had higher activity than sabinene. In contrast, the insecticidal activity of sabinene and 1,8-cineole was moderate and comparable; thus, the interaction was additive. The insecticidal activity of limonene was stronger than that of 4-terpineol and 1-octen-3-ol. However, 1,8-cineole had synergistic effects with 4-terpineol and 1-octen-3-ol, but an additive effect with limonene. This indicates that this might be due to the effect of compound types. Namely, for compounds with similar activity (IC_50_: 100–210 µg mL^−1^), the interaction between two terpenoids tends be synergistic. In contrast, if one is a terpene but the other is a terpenoid, the interaction tends to be additive. Nevertheless, if the activity of terpenoids is good enough, it is also possible for them to be synergistic with terpenes. For example, the activity of 4-terpineol, 1-octen-3-ol, or linalool is better than that of 1,8-cineole, and these compounds show synergistic effects with limonene.

## 4. Conclusions

The volatile compounds of BC-EO were identified and their composition was determined. Monoterpene hydrocarbons were the largest group among the identified compounds. Of them, α-terpinolene was the largest compound. We have developed a method to express the impact of individual compounds on the entire functionality of the EO. Regarding the interactive effects of test compounds, both additive and synergistic effects existed regarding the insecticidal activity. Many compounds found in the BC-EO are also widely present in other EOs. Thus, the information obtained from this study is useful for EO-related research or applications in selecting EOs or seeking the best combination of EOs or individual compounds to achieve efficient insecticidal effects.

## Figures and Tables

**Table 1:** Identification and composition of individual compounds in the BC-EO.

Compounds or Yield	Ordor Description ^a^ or Yield	RI ^b^	Identification ^c^	Composition (g kg^−1^)
α-Pinene	Woody	1052	MS, ST	7.62 ± 0.11
β-Pinene	Woody	1114	MS, ST	4.13 ± 0.04
Sabinene	Woody-herbaceous	1128	MS, ST	61.26 ± 0.52
Myrcene	Sweet, balsamic,e thereal-sweet	1163	MS, ST	7.75 ± 0.06
α-Terpinene	Herbaceous, citrusy	1156	MS, ST	8.67 ± 0.13
Limonene	Sweet-citrusy	1212	MS, ST	44.65 ± 0.24
1,8-Cineole	Citrus, herbaceous, minty	1226	MS, ST	177.99 ± 0.02
γ-Terpinene	Lemony-citrus	1260	MS, ST	196.24 ± 1.70
*p*-Cymene	Grassy-kerosene-like	1281	MS, ST	104.10 ± 0.91
α-Terpinolene	Plastic	1299	MS, ST	250.60 ± 1.45
1-Octen-3-ol	Earthy	1443	MS, ST	12.37 ± 0.01
Linalool	Floral-woody, citrus	1537	MS, ST	1.61 ± 0.01
4-Terpineol	Must-woody, warm-peppery	1605	MS, ST	40.02 ± 0.16
β-Caryophyllene	Spicy, woody	1618	MS, ST	15.26 ± 0.13
α-Terpineol	Lilac	1702	MS, ST	2.43 ± 0.23
Citronellol	Geranium, rose	1762	MS, ST	3.65 ± 0.21
β-Caryophyllene oxide	Sweet, spicy	2003	MS, ST	2.17 ± 0.04
Nerolidol	Woody-floral	2037	MS, ST	2.80 ± 0.03
Oil yield (g Kg^-1^ fresh wt)	0.11			943.2

^a^ As reported in the reference [16]. ^b^ Retention indices based on a DB-Wax column. ^c^ MS, identified by mass spectrum; ST, confirmed with a standard compound.

**Table 2:** Individual compounds of the *Bacopa caroliniana* essential oil (BC-EO) on acetylcholine esterase (AChE) inhibition and insecticidal activity.

Compound	AChE-IC_50_ (µL mL^−1^)	EI ^a^	Insect-IC_50_ (µg mL^−1^ Air)	TI ^b^
α-Pinene	1.81 ± 0.20	139.45 ± 2.07	282 ± 51	1.93 ± 0.03
β-Pinene	>500	-	778.07 ± 79.5	0.54 ± 0.00
Sabinene	85.03 ± 0.20	23.86 ± 0.20	391 ± 1.41	15.93 ± 0.13
β-Myrcene	>500	-	481 ± 12	3.18 ± 0.02
α-Terpinene	>500	-	546.21 ± 1.64	1.61 ± 0.03
Limonene	9.57 ± 1.37	154.54 ± 0.83	105 ± 22	6.23 ± 0.03
1,8-Cineole	31.13 ± 2.20	189.39 ± 0.02	328 ± 9	36.20 ± 0.00
γ-Terpinene	>500	-	839 ± 58.68	23.79 ± 0.21
*p*-Cymene	>500	-	961 ± 16.12	11.02 ± 0.10
α-Terpinolene	1.10 ± 0.17	7546.09 ± 43.65	172 ± 6	39.76 ± 0.23
1-Octen-3-ol	14.82 ± 0.12	27.65 ± 0.03	166 ± 5	7.58 ± 0.01
Linalool	44.33 ± 1.31	1.20 ± 0.01	149 ± 13	1.19 ± 0.01
4-Terpineol	11.29 ± 0.02	117.40 ± 0.48	188.60 ± 36.34	13.75 ± 0.06
β-Caryophyllene	>500	-	1017 ± 63	1.53 ± 0.01
α-Terpineol	18.39 ± 1.52	4.37 ± 0.42	207 ± 56	0.90 ± 0.09
Citronellol	86.08 ± 1.09	1.40 ± 0.08	269 ± 31	4.41 ± 0.25
Caryophyllene oxide	>500	-	>3000	-
Nerolidol	331.24±21.55	0.28 ± 0.00	699 ± 2.1	0.41 ± 0.00
Oil	1.205 ± 0.01	25929.21	205 ± 35.2	496.10

^a^ EI: the impact of AChE inhibitory activity. ^b^ TI: the impact of insecticidal inhibitory activity.

**Table 3:** Interactive effects of individual compounds on the AChE inhibition.

	α-Terpinolene	Sabinene	Limonene	α-Pinene	1,8-Cineole	4-Terpineol	1-Octen-3-ol	Linalool
α-Terpinolene	-							
Sabinene	0.5 ^a^ (S) ^b^	-						
Limonene	0.5 (S)	0.5 (S)	-					
α-Pinene	0.5 (S)	1 (A)	0.5 (S)	-				
1,8-Cineole	1 (A)	1 (A)	1 (A)	1 (A)	-			
4-Terpineol	1 (A)	0.5 (S)	1 (A)	1 (A)	0.5 (S)	-		
1-Octen-3-ol	1 (A)	1 (A)	1 (A)	1 (A))	0.5 (S)	0.5 (S)	-	
Linalool	1 (A)	1 (A)	1 (A)	1 (A)	1 (A)	1 (A)	1 (A)	-

^a^ Fractional inhibitory concentration (FIC) index. ^b^ synergy (FIC ≤ 0.5, S), addition (0.5 < FIC ≤ 1, A).

**Table 4:** Interactive effects of individual compounds on the insecticidal activity.

	α-Terpinolene	Sabinene	Limonene	α-Pinene	1,8-Cineole	4-Terpineol	1-Octen-3-ol	Linalool
α-Terpinolene	-							
Sabinene	0.5 ^a^ (S) ^b^	-						
Limonene	0.5 (S)	0.5 (A)	-					
α-Pinene	0.5 (S)	0.5 (S)	0.5 (S)	-				
1,8-Cineole	0.5 (S)	1 (A)	1 (A)	0.5 (S)	-			
4-Terpineol	0.5 (S)	0.5 (S)	0.5 (S)	0.5 (S)	0.5 (S)	-		
1-Octen-3-ol	0.5 (S)	1 (A)	0.5 (S)	0.5 (S)	0.5 (S)	0.5 (S)	-	
Linalool	0.5 (S)	0.5 (S)	0.5 (S)	0.5 (S)	0.5 (S)	0.5 (S)	0.5 (S)	-

^a^ FIC index. ^b^ synergy (FIC ≤ 0.5, S), addition (0.5 < FIC ≤ 1, A).
